# Enhancing understanding of interventions to increase relational continuity in general practice: a realist review protocol

**DOI:** 10.3399/BJGPO.2025.0119

**Published:** 2026-01-28

**Authors:** Serge Engamba, Jane Smith, Nada Khan, Kate Sidaway-Lee, Patrick Burch, Tom Marshall, Phil Evans, Denis Pereira Gray, Rob Anderson

**Affiliations:** 1 Exeter Collaboration for Academic Primary Care (APEx), University of Exeter, Exeter, UK; 2 St Leonard's Research Practice, Exeter, UK; 3 Division of Population Health, Health Services Research & Primary Care, The University of Manchester, Manchester, UK; 4 Department of Applied Health Sciences, University of Birmingham, Birmingham, UK

**Keywords:** general practice, primary health care, physician–patient relationship, continuity of patient care, realist approach, primary care delivery, quality of care, therapeutic relationships

## Abstract

**Background:**

Relational continuity of care (RCC), which is characterised by an ongoing therapeutic relationship between patients and their primary care providers, is critical for ensuring high-quality care in general practice. Despite its importance, challenges, such as staffing shortages, policy shifts, and evolving patient needs, often impede its consistent delivery. With the new GP contract in England highlighting the need for primary care providers to monitor and deliver relational continuity, it is more crucial than ever to understand how best to achieve it.

**Aim:**

To explore how, why, and under what conditions interventions to improve relational continuity are successfully implemented in general practice.

**Design & setting:**

The realist review will be supported by an expert stakeholder panel and a patient advisory group to consider the diverse and dynamic settings of general practice, and generate contexts, mechanisms, and outcomes configurations exploring how interventions to enhance RCC in general practice work.

**Method:**

Through the synthesis of diverse international evidence sources, including qualitative, quantitative, and mixed-methods studies, as well as grey literature, the review will develop an understanding of the mechanisms that produce relational continuity, the contexts in which these mechanisms operate, and the outcomes they produce for the health system, practices, practitioners, and patients.

**Conclusion:**

The findings will provide data to inform future research and refine strategies and policies that support the effective delivery of relational continuity, which in turn may lead to improved patient outcomes and enhanced care experiences.

## How this fits in

Relational continuity of care (RCC) is known to improve patient satisfaction, health outcomes, and reduce healthcare costs. However, the specific interventions that effectively enhance RCC in the context of modern general practice remain poorly understood. This research will elucidate how various interventions impact RCC, examining their effectiveness across different healthcare settings and patient populations. By providing evidence-based insights, this study will assist clinicians in implementing targeted strategies to strengthen RCC, ultimately enhancing patient care and system efficiency.

## Introduction

Relational continuity refers to the ongoing therapeutic relationship between patients and their primary care providers, enabling consistent, personalised care over time. It is recognised as a core component of high-quality general practice worldwide,^1^ associated with improved health outcomes, greater patient satisfaction, and more efficient healthcare use.^2–4^ However, delivering relational continuity is becoming increasingly difficult owing to growing healthcare system complexity, limited resources, and evolving policy priorities. Contributing factors include the rise of large super practices, a decline in full-time GPs, and the shift towards multidisciplinary teams. As a result, relational continuity is steadily declining, despite its well-established benefits.^5,6^

Although much is known about the value of relational continuity, less is understood about the mechanisms through which it is effectively achieved in varying contexts.^7,8^ A realist review is particularly well-suited to unpack this complexity by exploring what works, for whom, in what contexts, and why.^9^ The review will begin with a preliminary programme theory, developed through initial literature searches, theoretical insights, and consultation with key stakeholders. This theory will be iteratively tested and refined using data from a wide range of sources, with contributions from patient and public involvement and engagement (PPIE) and expert stakeholder (ES) groups. The final product will be a set of context–mechanism–outcome (CMO) configurations offering explanatory insights to inform future RCC interventions in general practice.

### Aim

This realist review aims to investigate how, why, and under what circumstances relational continuity of care (RCC) is achieved in general practice, and to explore the outcomes it produces for patients, healthcare providers, and the wider healthcare system.

### Research questions

- What interventions have been implemented globally to improve relational continuity in general practice, and what are their key components?
- How do these interventions work to enhance relational continuity in different primary care contexts?
- In what contexts are these interventions most or least effective in delivering relational continuity?
- What outcomes, both intended and unintended, are associated with interventions designed to improve relational continuity in general practice?
- How can interventions to improve relational continuity be tailored or adapted to different primary care settings to maximise their effectiveness?

## Method

A realist review is a theory-driven, interpretive approach to evidence synthesis that incorporates qualitative, quantitative, mixed-methods research, and grey literature. This review will focus on the UK and countries with comparable primary care systems, characterised by general or family practice leadership, multidisciplinary teams, group practice structures, autonomy in organisational decisions (for example, independent contractor status), and responsibility for defined patient lists. Focusing on similar systems broadens the scope and strengthens the relevance and applicability of the findings, ensuring that recommendations are contextually appropriate for UK general practice while offering insights beyond those available from UK-based evidence alone.

Using Starfield *et al*’s ‘4C framework’,^10^ a recent Organisation for Economic Co-operation and Development (OECD) and The Health Foundation report applied cluster analysis to group countries by healthcare system characteristics. We will draw on this report to identify countries within clusters 1 and 2, which share strong gatekeeping and continuity of care attributes.^11^

This study will follow Pawson’s five steps for realist reviews: (1) identifying existing theories; (2) searching for evidence; (3) selecting relevant studies; (4) extracting data; and (5) synthesising findings and drawing conclusions. The aim is to generate CMO configurations that explain how RCC is achieved. The review will follow the Realist And Meta-narrative Evidence Syntheses: Evolving Standards (RAMESES) quality standards.^9,12^

### Step 1: Locating existing theories

The first step involves identifying existing theories and frameworks that explain how RCC is achieved and sustained in general practice. This foundational work supports the development of an initial programme theory (IPT), which will guide the realist review. To inform this, we engaged our ES group — including researchers, GPs, and policymakers — and our PPIE group. These consultations provided valuable insights into current practice, perceived challenges, and assumptions about what enables or hinders RCC.

**Figure 1.**
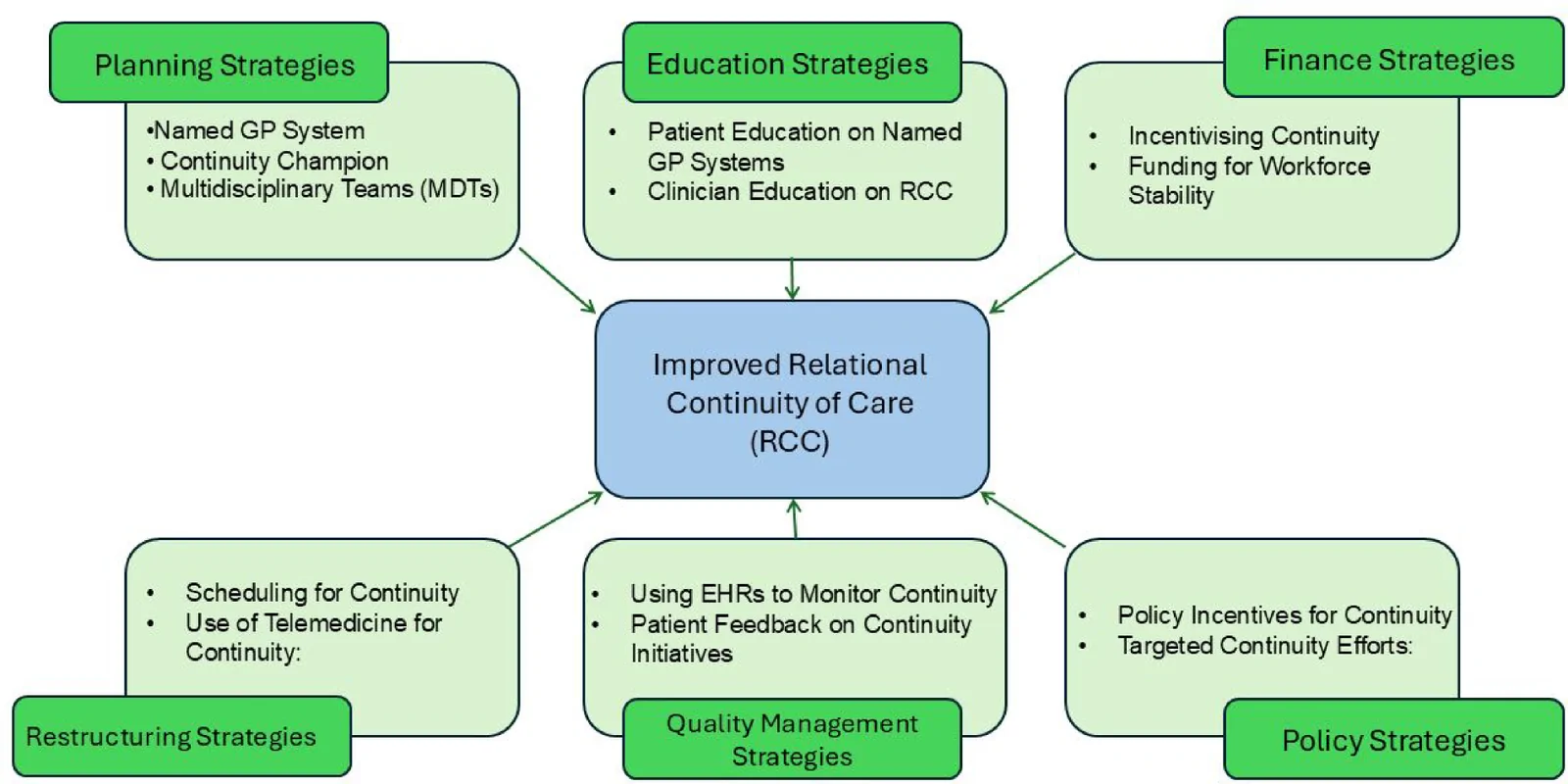
Initial programme theory: a synthesis of key initiatives. EHR = electronic health records. MDTs = multidisciplinary teams. RCC = relational continuity of care

Informed by this engagement, we reviewed key literature identified by the ES group, including the scoping review by Fox *et al* ^7^ and analytical articles by Pereira Gray *et al* ^13^ and Sidaway-Lee *et al*.^14^ These sources helped surface existing theoretical models and frameworks for RCC interventions (see Figure 1). Using this evidence and feedback from both the PPIE and ES groups, we developed and refined the IPT (Annexe 1), which outlines candidate mechanisms — such as relational, informational, and longitudinal continuity — and contextual factors, including practice structure, team composition, and patient characteristics.

The IPT also considers intended and unintended consequences, recognising that interventions may support RCC in some contexts but disrupt it in others. It will be iteratively tested and refined as the review progresses, supporting a nuanced understanding of how RCC functions across diverse settings.

### Step 2: Searching for evidence

We will search for literature to inform the development and refinement of the IPT created in step 1. Systematic searches will be conducted in MEDLINE, Embase, and CINAHL to identify peer-reviewed articles on RCC interventions in general practice. Search strategies will combine free-text terms and subject headings (for example, MeSH) such as ’relational continuity’, ’continuity of care’, ’general practice’, ’primary care’, ’patient–provider relationships’, and ’healthcare interventions’. No study design restrictions will be applied, allowing inclusion of qualitative, quantitative, and mixed-methods studies.

In addition to database searches, we will review the grey literature using Overton and organisations such as the NHS, Department of Health and Social Care, Royal College of General Practitioners (RCGP), The Health Foundation, and The King’s Fund, alongside relevant policy reports and conference proceedings. Citation tracking of key papers will be undertaken, and the PPIE and ES groups may suggest additional sources. This comprehensive strategy will ensure the capture of relevant interventions and outcomes. Results will be imported into EndNote21 for deduplication, then transferred to Rayyan software^15^ for title and abstract screening.

### Inclusion criteria

- Articles will be included if they provide information on the description, contexts, mechanisms, or outcomes of RCC interventions, specifically in UK general practice and in settings similar to UK primary care, according to the OECD and The Health Foundation report.^11^
- Articles published in English from inception of individual databases to 2024 will be included.

### Exclusion criteria

- Articles focusing on non-primary care settings.
- International studies pertaining to countries with systems that are significantly different from UK primary care.
- Studies that do not address RCC.

### Step 3: Article selection

Unlike narrative reviews and meta-analyses, realist reviews prioritise understanding generative causation through retroductive theorising over methodological quality.^16^ Following the literature search, a three-step screening process will be applied. First, reviewer 1 will screen titles for relevance. Remaining articles will then be assessed by title and abstract to ensure they address relational continuity in primary care. Rayyan software will support this screening. Where abstracts are unclear, full texts will be reviewed.

Reviewers 1 and 2 will screen all abstracts. To ensure inter-rater reliability, reviewer 3 will independently assess a random 10% sample. Reviewers 1 and 2 will then evaluate all relevant full texts for inclusion, based on their discussion of RCC, related interventions, or outcomes in general practice. The final set of included articles will be uploaded to NVivo (version 11) for storage and coding.

Disagreements or uncertainties about inclusion will be resolved through team discussion. Final inclusion will be guided by each document’s relevance, richness, and rigour in relation to the developing programme theory.^17^

### Step 4: Data extraction

Once the final set of articles is selected, full texts will be uploaded to NVivo (version 11) for coding. Data will be extracted inductively (emerging from the data), deductively (guided by the initial programme theory), and retroductively (to identify underlying causal mechanisms). The aim is to capture the contexts in which RCC interventions are implemented, the mechanisms through which they operate, and the outcomes they produce.^18^

The realist review is an iterative process, with searching, appraisal, and synthesis occurring concurrently and contributing to ongoing programme theory development. As the theory evolves, further data will be collected to test emerging concepts and uncover causal mechanisms, which may lead to refinement of previously extracted data.

Extracted data will include details of intervention components, provider and patient experiences, system-level impacts, and contextual factors affecting intervention effectiveness. Coding will also be iterative, adapting as evidence accumulates. As with article selection, a subset of data extraction decisions will be reviewed by two team members to ensure rigour and consistency.

### Step 5: Synthesising evidence and drawing conclusions

We will synthesise the extracted data using a realist logic of analysis to develop CMO configurations. These configurations will explain how and why RCC interventions succeed or fail in different contexts. For example, we will compare evidence from settings where RCC interventions have improved patient satisfaction and health outcomes with those where interventions have been less successful, aiming to understand the factors that contribute to these differing outcomes.

The synthesis will use cross-case comparison to explore the influence of various contextual factors, such as practice organisation, patient demographics, and the composition of multidisciplinary teams. The outcome of this analysis will be a refined programme theory, explaining how RCC interventions function in general practice and suggesting ways they can be tailored to different settings to maximise their effectiveness.^19^

Finally, the review will conclude with recommendations for practice and policy, offering actionable insights on how to design, implement, and adapt RCC interventions to improve relational continuity in general practice. The refined programme theory will guide these recommendations, and it will be shared with healthcare practitioners, policymakers, and researchers to enhance the sustainability and effectiveness of RCC interventions in primary care.

### Stakeholder and patient and public involvement and engagement (PPIE)

The review will engage GPs, patient representatives, policymakers, and administrators identified through expert panel recommendations. Stakeholders will help refine the programme theory and ensure the review addresses practical and policy-relevant concerns. Patients with and without experience of RCC will contribute, ensuring diverse perspectives. An equality of opportunity approach will guide the selection of public contributors to maximise involvement. PPIE group members will shape the programme theory, contribute to findings, and help develop accessible outputs such as documents and infographics. They will also play a key role in creating a dissemination strategy and ensuring solutions are both feasible and acceptable in real-world contexts.

## Discussion

### Summary

This realist review protocol is designed to explore the intricacies of how RCC is delivered in general practice, highlighting its critical role in improving patient outcomes and healthcare efficiency. This study is the first to use a realist approach to examine how interventions improve RCC, aiming to uncover the underlying mechanisms and contextual conditions that make these interventions effective. This approach offers deeper insights than traditional reviews by revealing how interventions, contexts, and outcomes interact.

### Strengths and limitations

The primary strength of this protocol lies in its comprehensive and theory-driven approach, which integrates diverse evidence sources, including qualitative, quantitative, and grey literature. This methodology enables a more detailed examination of the complex factors that influence the success of interventions aimed at enhancing RCC. The findings are expected to inform the development of targeted strategies that can be adapted to the specific needs and conditions of different primary care settings, potentially leading to more effective and sustainable improvements in patient care.

Despite its comprehensive approach, the study faces potential limitations. The focus on systems analogous to UK primary care may restrict the generalisability of the findings to other healthcare systems with different structures and patient engagement practices. Furthermore, excluding non-English language studies could omit valuable insights from diverse international contexts, potentially affecting the completeness of the evidence base. At this stage, the quality and depth of the literature remain uncertain, with many sources providing limited descriptions of interventions, while others lack clarity on outcome measures.

### Implications for research and practice

The anticipated findings of this review will inform both research and clinical practice. For researchers, it highlights the need to consider context and mechanisms when evaluating health interventions. For practitioners, it offers insights to support more effective, context-sensitive implementation of RCC. The results are expected to underpin future policy and practice guidelines to improve RCC delivery and patient outcomes in general practice.
